# Detection of anti-*Pneumocystis jirovecii* antibodies in human serum using a recombinant synthetic multi-epitope kexin-based antigen

**DOI:** 10.1007/s10096-020-03936-2

**Published:** 2020-06-16

**Authors:** Ana Luísa Tomás, Fernando Cardoso, Bruno de Sousa, Olga Matos

**Affiliations:** 1 Medical Parasitology Unit, Group of Opportunistic Protozoa/HIV and Other Protozoa, Global Health and Tropical Medicine, Instituto de Higiene e Medicina Tropical, Universidade Nova de Lisboa, Lisbon, Portugal; 2 CINEICC, Faculdade de Psicologia e de Ciências da Educação, Universidade de Coimbra, Coimbra, Portugal

**Keywords:** *Pneumocystis*, Pneumocystosis, Kexin protease, Synthetic recombinant antigen, Serological diagnosis, ELISA

## Abstract

Interest in the detection of specific anti-*Pneumocystis jirovecii* antibodies has emerged as less-invasive alternative diagnostic approaches. Here is presented the performance of an ELISA based on a recombinant synthetic multi-epitope kexin 1 (Kex1) antigen of *P. jirovecii*, previously developed. Results showed that IgM anti-Kex1 levels were found significantly increased in patients with *Pneumocystis* pneumonia (PcP) compared with non-PcP cases (*p* < 0.001), allowing a diagnostic performance of PcP with a 70.8% sensitivity and a 75.0% specificity. These results suggest that this Kex1-based ELISA is a promising tool toward the serodiagnosis of PcP when the standard methods are difficult to perform.

## Introduction

*Pneumocystis jirovecii* pneumonia (PcP) is still the most commonly diagnosed acquired immune deficiency syndrome defining disease in Europe [1]. Likewise, the rising number of other immunocompromised patients susceptible to *P. jirovecii* infection [2] warrants the need for improved disease management strategies. Nowadays, PcP diagnosis still relies on microscopic visualization of the organisms or their DNA detection in specimens obtained by invasive and expensive techniques, difficult to perform in respiratory failure patients, in children, and in resource-limited settings [3, 4]. Therefore, an alternative diagnostic approach is required.

The interest in serum antibodies as alternative tools for PcP diagnosis has increased since the demonstration of the humoral immunity important role in disease resolution [5–8]. *Pneumocystis* major surface glycoproteins (Msg), as its most abundant cell surface proteins, were the obvious candidates to start studying serological responses against PcP [9–14]. However, Msg’ variability [15] may compromise the accuracy of serological tests when recombinant antigens of this protein are used. This limitation may be the reason for the low sensitivity (68.0%) and specificity (61.8%) of a previously developed Msg-based ELISA [14].

Therefore, new antigenic candidates have been explored. Reports of high human antibodies’ titers to a recombinant subunit of *Pneumocystis* kexin-like serine protease (Kex1) correlated with a reduced incidence of PcP [13], protection against acquisition of *Pneumocystis* infection by vaccination with recombinant Kex1 peptides in immunosuppressed non-human primates [6], and the fact that Kex1 holds an antigenically stable active site sequence coded by a nuclear single-copy gene [16, 17] confirmed the interest in this protein. Thus, a newly recombinant synthetic (multi-epitope) antigen (RSA) based on the immunogenic behavior of *P. jirovecii* Kex1 was designed, produced, and applied in the development of a promising lateral flow immunoassay (LFIA) for PcP diagnosis [18]. However, the diagnostic performance of a Kex1-based ELISA is missing in order to understand if this new Kex1 RSA shows any diagnostic advantage over the previous Msg RSA. Therefore, we used IgG- and IgM Kex1-based ELISA to study sera from HIV-infected patients with and without PcP, in order to assess and discuss their applicability in PcP serodiagnosis.

## Material and methods

The design and purification process of the Kex1 RSA used in this study was previously described [18].

This was a retrospective observational study that included 76 sera from HIV-infected patients with a clinical picture of pulmonary disorders. Forty-eight sera samples were from patients with active PcP (positive laboratory detection of *P. jirovecii*) and 28 from patients with pneumonia due to other causes (negative laboratory detection of *P. jirovecii*), according to patient’s categorization detailed in Tomás et al. 2019 [18]. The morbidities associated with pulmonary symptoms in patients without *P. jirovecii* infection were not known at the time of the patient’s enrollment.

The Kex1 RSA was applied as antigenic tool in an indirect ELISA to detect IgG and IgM anti-*P. jirovecii* in patients’ sera. All volumes used were of 50 μL/well if not otherwise mentioned. Microplate odd-numbered columns were coated with Kex1 RSA (5 μg/mL in carbonate buffer pH 8.4) and even-numbered columns with PBS 1x (control), overnight at 4 °C. Plates were washed with washing buffer (PBS with 0.05% Tween-20) and blocked with blocking buffer (1% polyvinyl alcohol, 70 μL/well) for 1 h at 20–25 °C. The blocking buffer was removed, and serum was added to each well with a specific dilution concerning the immunoglobulin to detect (1/20 dilution in washing buffer for IgM and 1/40 dilution in washing buffer for IgG). Plates were incubated (1 h, 37 °C) and washed three times with washing buffer and one time with distilled water. An alkaline phosphatase-labeled monoclonal antihuman immunoglobulin G (A2064, sigma®) or M (A2189, sigma®) was added to each well (1/3000 or 1/1000 dilution in washing buffer, respectively) for 1 h at 37 °C. After repeating the washing steps, 4-nitrophenylphosphate sodium salt (1 mg mL^-1^) was added. Color developed overnight at 4 °C, and reading was performed at a wavelength of 405 nm (Infinite 200 Pro, Tecan®). ELISA results were determined for each specimen in duplicate in even- and odd-numbered columns. The mean of the even-numbered wells was deducted from the mean of the odd-numbered wells to obtain the final read for each sample.

Statistical analysis was performed at 0.05 significance level using the IBM SPSS version 20.0 and the open source R software version 4.0.0 [19, 20]. The non-parametric Mann-Whitney *U* test was used to examine the differences between the distribution of antibodies’ titers in patients with and without PcP. The Cohen’s d effect size for both IgG and IgM tests was calculated based on sample size of 48 PcP patients and 24 non-PcP patients and a significance level of 5% with the effsize package version 0.8.0 [21] from R Project software [20]. Receiver operating characteristic (ROC) curves, sensitivity, specificity, positive/negative predictive values, and likelihood ratios of the ELISA were calculated using the R package “optimal cut-points” [19]. With the “MaxSpSe” criteria of this package, optimal cut-off values were calculated [22, 23], to simultaneously maximize the sensitivity and specificity of the ELISA.

## Results

**Fig. 1:**
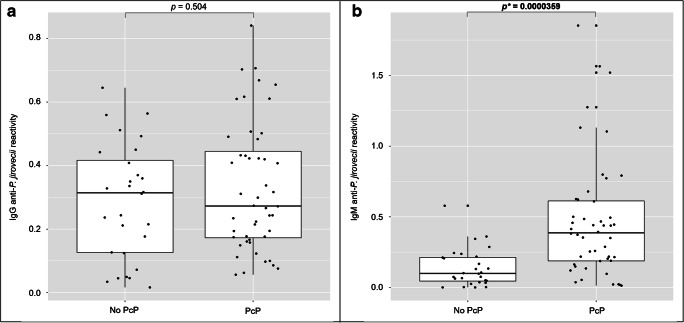
Simple boxplots showing the reactivity levels (OD at 405 nm) of IgG (**a**) and IgM (**b**) anti-*P. jirovecii* antibodies detected by ELISA protocols applied with the Kex1 RSA across patients with PcP and patients with pneumonia due to other cases (no PcP). The statistic values (p*), representing a statistically significant difference from Mann-Whitney *U* tests performed between the groups, are highlighted

**Fig. 2:**
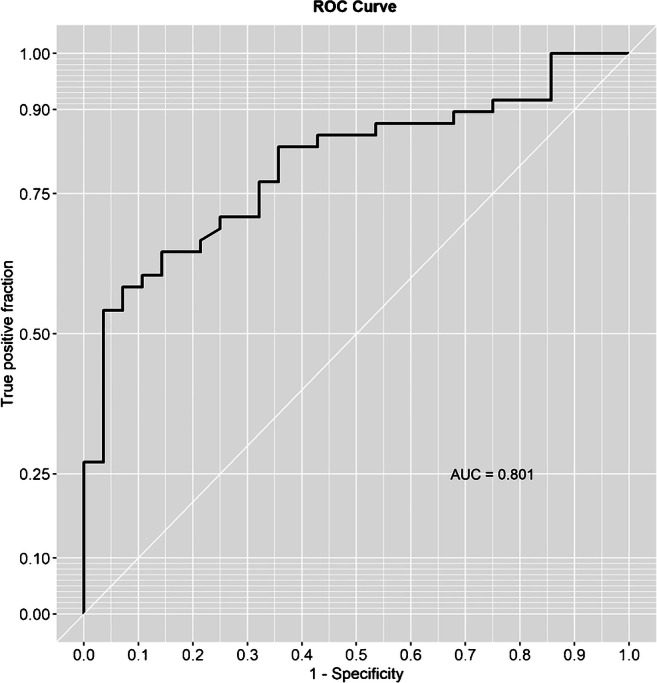
Representation of Kex1-based IgM ELISA ROC curve

ELISA was developed for detection of specific IgG and IgM anti-*P. jirovecii* antibodies in sera, applying the Kex1 RSA as antigenic tool. The distribution of the IgG and IgM anti-*P. jirovecii* reactivity levels across PcP and non-PcP patients is presented in Fig. 1. The median reactivity levels of the IgG anti-*P. jirovecii* detected in PcP patients were not statistically different (*p* = 0.504) from the levels detected in patients with pneumonia due to other causes. However, IgM anti-*P jirovecii* median reactivity levels were statistically higher in PcP patients (0.3871) compared with non-PcP patients (0.0997), showing applicability in the discrimination of these two groups of patients. Although a moderate sample size, the computed achieved power for IgG and IgM was 94.5% and 76.5%, respectively, based on a Cohen’s d of − 0.858 for IgG and − 0.646 for IgM. The assay ROC curve is represented in Fig. 2, showing an area under the curve of 80.1%. Optimal cut-off values were determined for PcP diagnosis based on IgM anti-*P. jirovecii* reactivity levels, and a value of 0.2149 was determined as the best to maximize both the specificity and the sensitivity of the assay. The diagnostic performance of the developed Kex1 RSA-based IgM ELISA is presented in Table 1 and indicates a sensitivity of 70.8%, specificity of 75.0%, and positive and negative predictive values of 82.9% and 60.0%, respectively.

**Table 1:** Accuracy measures of the Kex1 RSA-based IgM ELISA for PcP diagnosis

Kex1 RSA-based IgM ELISA	
Sensitivity	70.8%
(CI)	(55.9–83.0%)
Specificity	75.0%
(CI)	(55.1–89.3%)
Positive predictive value	60.0%
(CI)	(66.5–90.7%)
Negative predictive value	82.9%
(CI)	(43.9–80.7%)
Positive likelihood ratio	2.83
(CI)	(1.45–5.52)
Negative likelihood ratio	0.39
(CI)	(0.24–0.63)

## Discussion

PcP diagnosis is currently based on microscopic visualization of *P. jirovecii*, using cytochemical or IF-Mab staining, and/or detection of its DNA in the affected tissues [3, 4]. The gold-standard biological specimens for these tests (bronchoalveolar lavage or induced sputum) are obtained by invasive and expensive techniques, not always available or feasible to perform in patients with respiratory failure, in children, or in countries with limited resources [3, 4]. Therefore, the demand for an alternative using less demanding technologies and minimally invasive biological specimens directed attention and interest to blood and serum.

Although it has been demonstrated the applicability of recombinant antigens of *P. jirovecii* proteins in seroprevalence studies [10–13], their application in the development of a less-invasive approach for PcP diagnosis is just in the initial stage [12, 14, 18]. Recently, our research group designed and developed an Msg RSA-based ELISA that proved to have application as a serological approach for PcP diagnosis [14]. Though, its performance was not as good as intended, possibly due to *P. jirovecii* evasion mechanism based on Msg antigenic variation [15]. Thus, we produced a new RSA based on the immunogenic behavior of *P. jirovecii* Kex1 protein. The idea was to create an RSA from a single-copy protein [17] with an important role in disease protection [6, 13], avoiding the genetic variation feature that Msg exhibits [15].

As previously demonstrated [18], this new Kex1 RSA showed applicability in the detection of specific IgM anti-*P. jirovecii* antibodies when applied as an antigenic tool in LFIA techniques to study active PcP in human sera. However, this brief research note aims to present the diagnostic performance of a Kex1-based ELISA and discuss their implications for future guidelines for new diagnostic studies in the area of *Pneumocystis* pneumonia. For that purpose, the detection of IgG and IgM anti-*P. jirovecii* antibodies was performed in sera of 48 patients with active PcP and 28 patients with pneumonia due to other causes.

With the Kex1 RSA-based IgG ELISA, it was not possible to distinguish PcP and non-PcP patients (Fig. 1). An achieved power of 94.5% supports that the non-statistically significant differences found between IgG levels of PcP and non-PcP patients are not trivial. This result was also observed with the Msg RSA [14, 18] and may be explained by the previous colonization with *P. jirovecii* of patients presenting diverse levels of immunodeficiency, primary respiratory disorders, or even in the immunocompetent general population [8, 24, 25]. In contrast, the Kex1 RSA-based IgM ELISA showed successful application in discriminating PcP and non-PcP patients (Fig. 1), since IgM levels were detected significantly increased in patients with PcP (*p* < 0.0001). These results corroborate our previous findings with the Msg RSA and what was verified in studies with mice, which suggest that the IgM isotype has a predominant role in shaping the earliest steps in the recognition and clearance of *P. jirovecii* infection [14, 26]. However, it should be noted that this Kex1 RSA-based IgM ELISA presented a higher sensitivity (from 68.0 to 70.8%) and specificity (from 61.8 to 75.0%) for PcP diagnosis compared with our previous Msg RSA-based IgM ELISA, with a moderately high positive diagnostic likelihood ratio (2.83) and low negative diagnostic likelihood ratio (0.39) (Table 1). This was also consistent with preliminary results obtained with both Kex1 and Msg LFIA developed [18] and possibly can be explained by Msg genetic variation during infection, which could lead to false-negative results and a lower capacity of Msg RSA to detect anti-*P. jirovecii* antibodies in all disease phases.

With this brief research report, we show that this new Kex1 RSA-based ELISA has a high diagnostic potential and reinforces the idea that RSA is one of the most promising tools to achieve routine PcP serodiagnosis. We hope that this will support the development of new studies focused on Kex1 RSA-based strategies, in order to validate and optimize a simpler, faster, and less invasive diagnostic solution to overcome current diagnostic challenges, especially in resource-limited settings.
